# Bioelectroanalytical Technologies for Advancing the Frontiers To Democratize Personalized Desired Health

**DOI:** 10.1021/acs.analchem.5c01450

**Published:** 2025-05-14

**Authors:** Susana Campuzano, Víctor Ruiz-Valdepeñas Montiel, Rebeca M. Torrente-Rodríguez, José M. Pingarrón

**Affiliations:** † Departamento de Química Analítica, Facultad de CC. Químicas, Universidad Complutense de Madrid, Pza. de las Ciencias 2, Madrid 28040, Spain; ‡ CIBER of Frailty and Healthy Aging (CIBERFES), Instituto de Salud Carlos III, C/Monforte de Lemos 3-5, Madrid 28029, Spain

## Abstract

The breakthroughs experienced in the development of cutting-edge, reliable, and multipurpose (bio)­electroanalytical technologies and their successful incursion into underexplored scenarios have demonstrated their unique potential to act as enablers of the ongoing transformation from reactive to predictive, preventive, personalized, and participatory healthcare, currently known as “P4” medicine. This transformation, more than a vision, is a necessity to achieve a new generation of more efficient, sustainable, and tailored to individual needs healthcare. This promising outlook is the focus of this Perspective, which in addition to highlighting some of the most shocking related research over the last 5 years, offers a prospective and insightful view of the opportunities and imminent advances that shape the future of these fascinating technologies.

## Graphical abstract

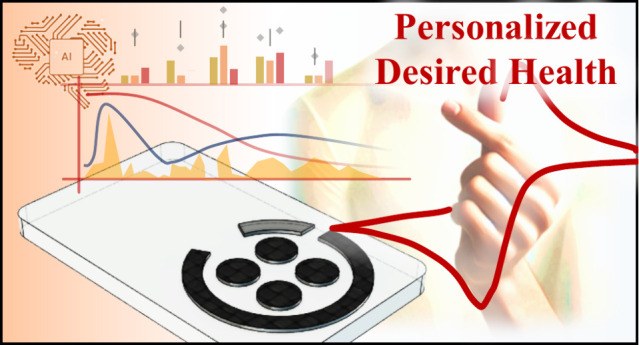

## Bioelectroanalytical Biotechnologies and Personalized Health

Precision medicine is transforming healthcare by pioneering procedures that improve prevention, diagnosis, treatment, and rehabilitation. Using cutting-edge therapies and innovative medicines, this approach personalizes care, leveraging science, digitization, and innovation to meet the challenges of modern healthcare.^1,2^ Personalized nutrition, closely linked to precision medicine, is an emerging paradigm which adapts dietary recommendations to the genetic profile of each individual. It optimizes the intake of nutrients and functional foods, while considering sustainability and environmental responsibility.^3^

To truly democratize the personalized desired health, which integrates precision medicine, therapeutics and nutrition, a bold, interdisciplinary strategy is required. This makes the collaboration of scientists and clinicians essential, united by their expertise and commitment to identify new (bio)­markers and develop sustainable next-generation technologies. The goal is clear: to bridge the gap between research and real-world application, transferring innovative discoveries from laboratories to clinical practice and society. It is to this high-stakes landscape that research focused on creating innovative multiplexed, multi-omics, multi-purpose and fully integrated electroanalytical (bio)­technologies are addressing to empower precision medical care. These advances serve as fundamental enablers towards a future in which medicine, therapy and nutrition are not only personalized, but also accessible to all.

Recent developments have highlighted the immense potential of (bio)­electroanalytical technologies to drive both research and real-world applications in precision medicine and nutrition.^4–6^ These innovations have thrived by adopting a bold and collaborative approach, seamlessly integrating the advances in electrochemical biosensing^7,8^ with those experienced also by other cutting-edge technologies. Figure 1 illustrates this synergy and shows the powerful partnerships that, together with electroanalytical biotechnologies, are shaping the future of healthcare. These key technological advances and their role in driving the next generation of precision medicine and nutrition are briefly discussed below.

- Versatile electrode substrates in terms of use (reusable or disposable^9,10^ ), manufacturing materials (paper,^11^ food or ingestible products,^12^ plastic, textile, polymeric, tattoo), and properties (superwettable,^13^ flexible and stretchable^14^) and the progress in miniaturized bioelectrochemical electronics^15^ that have been seamlessly combined in new electrochemical sensor formats: wearables,^16^ implantable,^17^ microneedle-based,^18,19^ etc. Notably, the evolution of microneedle sensors for dermal interstitial fluid analysis is changing the landscape of biodetection.^19^ A similar transformation is the integration of nucleic acid-based electrochemical sensors^20^ and aptamers^21^ into implantable and portable platforms, ushering in a new era of precision medicine and personalized healthcare. Nucleic acid-based biodetection is recognized as a frontier of vast untapped potential, opening exciting new opportunities for next-generation diagnostics and treatment strategies.^22^
- Microfabrication and microfluidics technologies, which have been leveraged for the development of microfluidic,^23^ lateral flow assay (LFA)-based^24^ and organ-on-chips (OoC)^25^ electrochemical biodevices. These advances bridge the gap between current *in vitro* and *in vivo* models and play a crucial role in drug discovery and disease pathophysiology research. They are also essential for the development of fully integrated electrochemical biodetection platforms, which have significant potential for commercialization in the point-of-care (POC) diagnostics market.^26^
- Cutting-edge omics technologies (quantitative and targeted proteomics and immunomics), which are key for the identification of new targets and potential receptors for their determination.^27^
- Nature-inspired (bio)­mimetic receptors^28^ that include a variety of innovative biomolecules, such as aberrant and/or phage-presented peptides,^29^ alternative splicing proteoforms,^30^ ectodomains derived through targeted mutations,^31^ complete proteins produced in cell-free or mammalian systems,^32^ cell membranes,^33,34^ molecular switches^35^ and inverted molecular pendulums.^36^
- Chemical functionalization and immobilization strategies that are attractive in terms of sensitivity, simplicity, multiplexing and/or reusability (“click” or electrografting,^37^ His-tag^38^ and HaloTag^27,29,30,39^ chemistries).
- Artificial and biological (nano)­materials (with electrocatalytic, *pseudo*-enzymatic, biocompatibility properties and carrying a multitude of functional groups)^40^ for the modification of electrode surfaces^41,42^ and the preparation of signaling nanotags.^37,42^

- Commercial reagents with multiple tracers or enzymatic molecules^43^ to amplify the electrochemical response.
- Ratiometric strategies that effectively overcome the background noise problems inherent to electrochemical sensors by using the signal ratio of multiple electrochemically active substances, thus improving the accuracy and repeatability of measurements.^44^
- Strategies for isothermal nucleic acids amplification, such as reverse polymerase amplification (RPA), loop-mediated isothermal amplification (LAMP), exponential amplification reaction (EXPAR), and primer-exchange reaction (PER).^45–47^

- Gene-editing technologies, improving detection limits and accuracy to detect both genetic and nongenetic targets with high efficiency using simple and rapid approaches.^48,49^

- State-of-the-art statistical and artificial intelligence (AI) tools to improve the accuracy, efficiency and accessibility of electroanalytical biotechnologies, especially in complex environments and diverse biological samples. These advances are driving smarter, faster and more reliable diagnostics.^2,50,51^

**Figure 1:**
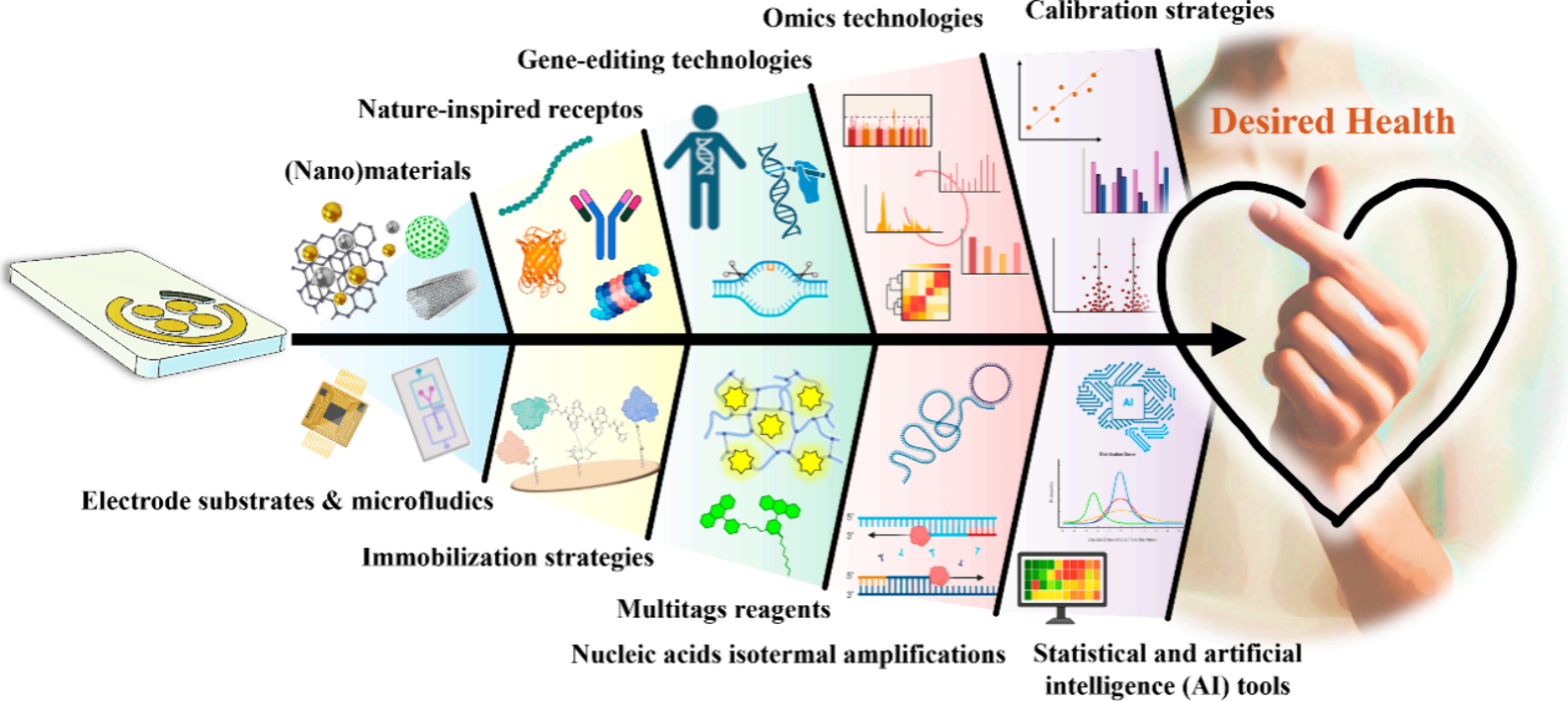
Cutting-edge (bio)­electroanalytical technologies and their key partnerships (created with biorender.com).

The integration of modern (bio)­electroanalytical technologies and their strategic alliances represents bold, powerful and synergistic innovations. These collaborations have demonstrated their unique competitive advantages over traditional and state-of-the-art alternatives. They offer unmatched simplicity, cost-effectiveness, design versatility and adaptability in diverse environments. Such technologies are especially useful for exploring and validating emerging biomarkers, whether identified by advanced omics technologies or by computational modeling, while also tackling the challenge of limited market availability of bioreceptors and standards. Recent advances have demonstrated the remarkable compatibility of electrochemical biodetection with the simultaneous or individual analysis of biomarkers at various molecular levels, ranging from genetic and epigenetic to proteomic and metabolomic. Their versatility allows the analysis of a broad spectrum of samples, including biological fluids (blood, plasma, serum, sweat, urine, saliva, tears), exhaled air, cells (lysed or intact), exosomes, tissues and even plant and animal organelles. All in all, these technologies are constantly opening new possibilities for in-depth studies in increasingly complex matrices and scenarios.

In the field of personalized nutrition, significant progress has been made in the development of biotechnologies to determine molecular markers in biofluids. Notably, these platforms operate decentralized or directly in the body, such as the analysis of stimulated sweat, offering innovative approaches for personalized medical care in real time.^52,53^

Despite the wide range of markers that influence individualized nutrition (from essential amino acids and vitamins to sugars and specific immunoglobulins), most electrochemical bioplatforms targeting dietary biomarkers in body fluids have focused on vitamins. However, given the intrinsic link between nutrition and health, biotechnologies currently used in clinical settings to measure markers such as glucose, ketone bodies, ferritin, and cholesterol also hold great promise for applications in personalized nutrition.^6^

Modern electroanalytical biotechnologies are designed in integrated formats on various electrode substrates and in innovative configurations that leverage the benefits of various magnetic microcarriers, such as microparticles^54^ and micromotors^55–57^ (Figure 2). The versatility of these microcarriers in fabrication and functionalization enables bioassays with increased sensitivity, reduced reagent and sample requirements, and accelerated kinetics, thanks to controlled agitation of microparticles or autonomous movement of micromotors in the presence of suitable fuels. Furthermore, by decoupling sample analysis from detection steps, these strategies effectively mitigate interference from complex matrices, setting a new standard for robust and efficient bioanalysis. Although micromotors do not yet have a monopoly on magnetic microparticles in bioelectroanalytical technologies due to their later incorporation, because of the lack of commercial availability, their intrinsic versatility, especially those fuel-free and made of biocompatible or biodegradable materials, coupled with their ability to move autonomously, makes them particularly attractive when sample volume is severely limited and/or for point-of-need applications, where the incubators that requires the magnetic microparticles handling can be considered a disadvantage.

**Figure 2:**
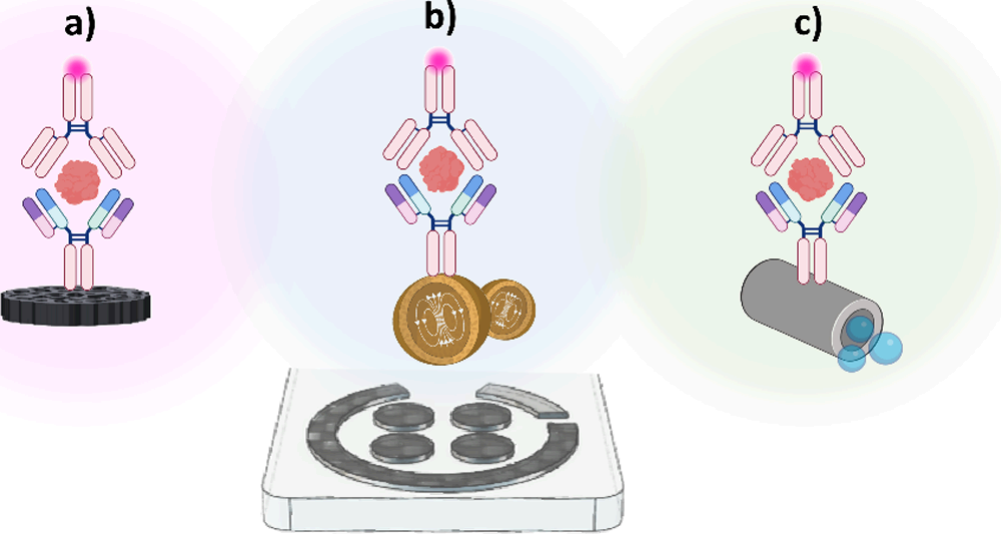
Schematic diagram of electroanalytical technologies based on sandwich immunoassay formats developed in integrated formats (a) or assisted using different supports [magnetic microparticles (b) and micromotors (c)] (created with biorender.com).

Regarding electrochemical transduction, bioelectroanalytical tools predominantly involve amperometry and voltammetry, although they also use electrochemical impedance spectroscopy (EIS) and electrochemiluminescence (ECL). Amperometric and voltammetric techniques are renowned for their exceptional sensitivity, affordability, fast response times, ease of miniaturization, automation and seamless integration into continuous analysis and point-of-care (POC) configurations. EIS, recognized for its ability to examine interface properties (including biorecognition events on electrode surfaces),^58^ is a powerful method, but comes with complexities. It requires specialized expertise for data interpretation, suffers from longer measurement times, and faces challenges in data acquisition at very low frequencies. ECL, which synergistically combines electrochemical and chemiluminescent strategies, offers the best of both worlds: high selectivity, minimal background interference, fast responses and a wide detection range. However, despite its promising attributes, ECL is still in its early stages of adoption and instrumentation compared to long-established pure electrochemical techniques.^59,60^

Due to the interest aroused by electroanalytical biotechnologies in the field of health, the number of related publications has increased almost exponentially in the last decade (Figure 3, with 465 publications since 2014, 77.8% of them in the last 5 years). Interestingly, review articles covering general^61^ and specific aspects, such as the analysis of whole blood,^62^ POC testing (POCT),^63^ and cancer diagnosis,^64^ have been recently reported.

**Figure 3:**
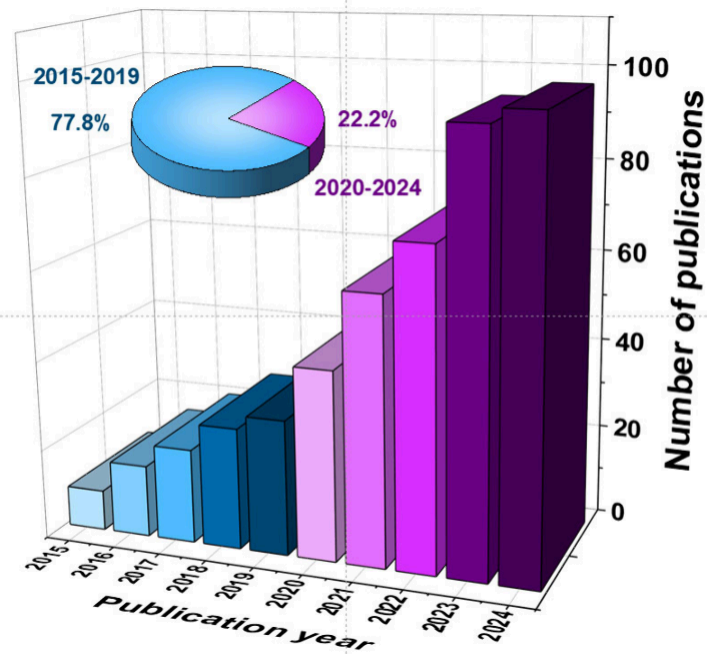
Number of publications resealed in the last decade according to Web of Science using the search terms “electrochemical biosensors” and “health”. Inset: percentage of the total number of these publications in each five-year period.

However, driven by the stringent demands of democratizing optimal health, multi-target and multi-purpose electroanalytical technologies are currently emerging as highly attractive solutions. Equally compelling are innovations that venture into previously unexplored scenarios or pioneering applications in precision medicine and personalized nutrition and those focused on developing fully integrated electrochemical biosensing technologies, bridging the gap to translation and commercialization. This is why this perspective article, unlike other recent reviews, aims to highlight the advances experienced in the development of cutting-edge, multi-objective and multi-purpose (bio)­electroanalytical technologies and their successful foray into little-explored scenarios to democratize the desired personalized medical care, also offering a personal, critical and futuristic vision of this exciting topic. With this intention, the following sections explore the unique appeal of these technologies, giving a broad overview, without delving into the details, of the challenges they have overcome, while highlighting the exceptional opportunities they have. This is done through a small, selected sample of the most striking 2020 developments, culled from the large and diverse collection that defines the state of the art.

### Multitarget Electroanalytical Technologies

The inherent complexity and variability of diseases such as cancer imply that electrochemical biotechnologies that focus on a single analyte often lack the necessary specificity and sensitivity, leading to the risk of false positives or negatives.^64^ On the other hand, since disease progression is related to a complex network of biomolecules, it is widely accepted that the evaluation of a broad panel of biomarkers allows for more accurate diagnoses and personalized treatments. Furthermore, the ability of electrochemical biotechnologies to simultaneously detect multiple classes of biomolecules in a single assay not only maximizes the information gained from limited reagents and sample volumes but also improves efficiency and speeds up the diagnostic process.^65^ These capabilities make electroanalytical biotechnologies particularly valuable as powerful tools for the accurate diagnosis, prognosis and therapeutic monitoring of complex and heterogeneous diseases, such as cancer, cardiovascular disorders, infectious and neurological diseases, at the point-of-need and/or in resource-limited settings. However, it is important to recognize that the electrochemical determination of multiple analytes is a complex task, whether they are of the same or different molecular level. Multiplexed detection and determination often require the use of different tracers on a single electrode or the implementation of electrode arrays when a common tracer is used.^66^ In addition, biomarkers, even those at the same molecular level, can exist in very different clinical ranges, and their simultaneous detection and determination often require diverse bioreceptors and assay formats. This variability is further influenced by the commercial availability or in-house production of these bioreceptors. Fortunately, the inherent versatility of electroanalytical technologies has successfully overcome most of these challenges. For example, innovative devices now integrate multiple types of bioreceptors, such as peptides and full-length proteins produced in mammalian cells for the detection of specific antibodies.^32^ In addition, they can combine immunoassay formats with various enzymatic labeling strategies^67^ or merge different bioassay strategies, such as enzymatic assays with immunoassays^68^ or enzymatic with aptasensing assays also employing different transduction techniques^69^ (Figure 4). The potential of electroanalytical technologies to facilitate comprehensive, multiplexed analyses demanded in the expanding landscape of precision medicine and personalized nutrition is clearly highlighted by all these advances.

**Figure 4:**
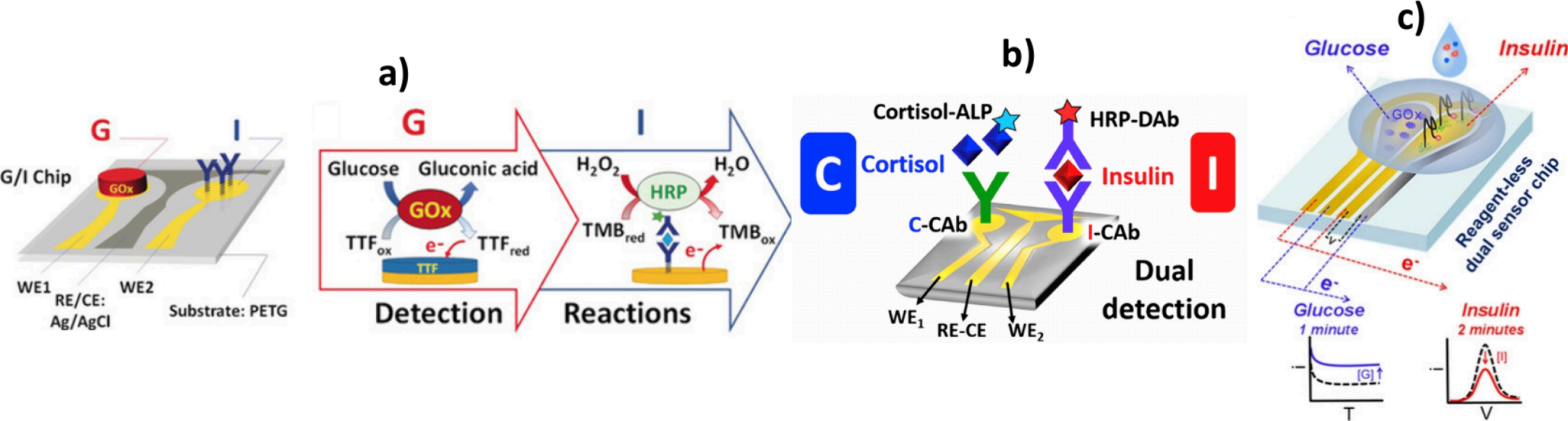
Versatility of electrochemical biotechnologies to integrate various bioreceptors, bioassays, immunoassay formats and transduction techniques into a single device. Schematic diagrams of individual chips that integrate an enzyme assay with an immunoassay (a), different immunoassay formats and enzyme tracers (b), and an enzyme assay with an aptaassay using different strategies for transduction (c). (a) Reprinted with permission from ref 68. Copyright 2019 Wiley-VCH. (b) Reprinted with permission from ref 67. Copyright 2020 Elsevier. (c) Reprinted with permission from ref 69. Copyright 2024 ACS.

To improve diagnostic accuracy and therapeutic outcomes, electroanalytical biotechnologies can be designed to simultaneously detect multiple biomarkers at various molecular levels. This multitarget approach is particularly transformative for the management of complex diseases, such as cancer, neurodegenerative diseases, autoimmune diseases, and viral infections, by yielding a more detailed snapshot of patient’s health conditions. Unlike conventional techniques, such as PCR or single-analyte immunoassays that detect only one class of biomolecule, these technologies offer the fundamental advantage of measuring several classes of biomolecules at once.^65^ Recent advances have pushed the boundaries of multiplexing: while most current systems detect 2–4 markers,^70^ some emerging technologies now simultaneously measure 8 markers,^32^ and recent innovations even allow the simultaneous detection of 96 biomarkers through integrated photoelectrochemical (PEC) detection.^71^

Diverse technologies have been designed to determine multiple targets of the same molecular type to identify tumor-associated nucleic acids (both DNA and RNA) and protein markers, as well as epigenetic modifications in nucleic acids.^70,72^ These platforms also enable the analysis of extracellular vesicles, tumor cells (including their surface proteins and cargo) and the assessment of autoantibodies in cancer, autoimmune, neurodegenerative and infectious diseases.^27,29–32,39,73^ With the advancement of electroanalytical tools, researchers have opened the door to more personalized diagnostics, as well as exploring the clinical role of antibody isotypes in early detection, prognosis, and disease burden assessment.

Among the biotechnologies developed to detect simultaneously different classes of (bio)­molecules,^64,65^ which are still relatively rare, some notable advances are the simultaneous detection of ctDNA and tumor protein markers;^74^ viral nucleic acids or viral proteins together with host IgG antibodies^75,76^ or therapeutic antibiotics;^77^ and the simultaneous analysis of a protein and its associated mRNA.^78^ These pioneering platforms, some illustrated in Figure 5, provide a broader view of evolving disease and therapeutic responses and break down barriers in diagnostics and personalized medicine.

**Figure 5:**
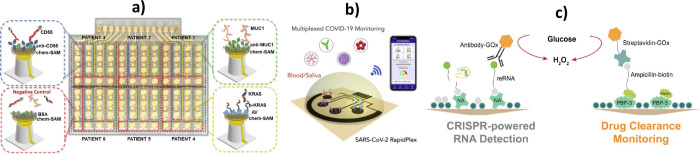
Multitarget electroanalytical technologies developed for the simultaneous determination of ctDNA and tumor protein markers (a), viral proteins and host IgG antibodies (b), and viral nucleic acids and therapeutic antibiotics (c). (a) Reprinted with permission from ref 74. Copyright 2023 Wiley-VCH. (b) Reprinted with permission from ref 75. Copyright 2020 CellPress. (c) Reprinted with permission from ref 77. Copyright 2022 Elsevier.

Due to their inherent characteristics, other electroanalytical technologies can be easily customized to detect targets at various molecular levels, they being of particular interest for multiplexed analysis at different molecular levels. A noteworthy example is the method published by Gong et al., which is based on host–guest interactions between methylene blue (MB)-labeled probes and β-cyclodextrin (β-CD)-based nanocomposites.^79^ This strategy made it possible to achieve the sensitive detection of p53 DNA, microRNA-21, and thrombin protein.

### Multipurpose Electroanalytical Technologies

Because of their inherent principles and the diversity of the targets they measure, some electroanalytical technologies have been classified as multipurpose. These technologies, depending on the interest, can be designed to detect a single marker or multiple markers, and offer versatile solutions for a wide range of diagnostic and therapeutic needs.

In the former group, electroanalytical technologies designed to measure ion channels (transmembrane proteins that regulate ion flow, such as potassium channels involved in the development of cancer cells) can be mentioned as an example. The developed methodology not only allows the assessment of the activity of specific ion channels, but also facilitates the identification of potential blocking agents, such as polypeptides derived from the venom of certain scorpion species (Figure 6a).^80^ The fundamental nature of the developed bioplatforms allows easy adaptation to detect different ion channels in the same cell type or in several cell types, simply by changing the capture and detection antibodies. This versatility makes them a promising tool for advancing the understanding of channelopathies (which include, in addition to cancer, Alzheimer’s disease and multiple sclerosis) and improving the efficacy of ion channel therapies.

**Figure 6:**
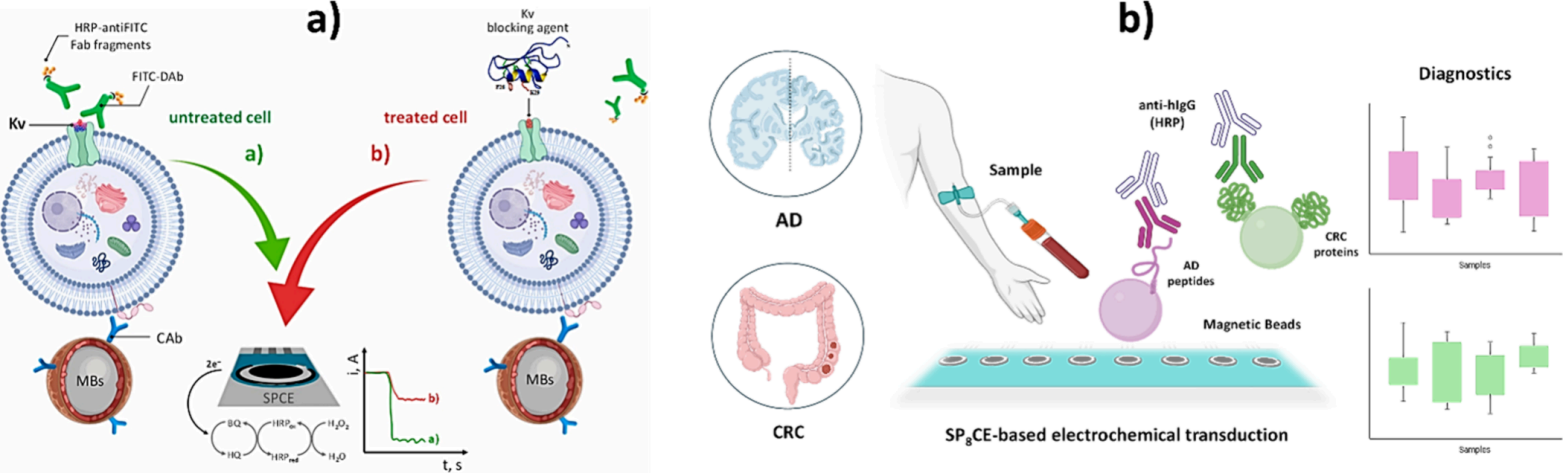
Multipurpose electroanalytical technologies to assess the activity of specific ion channels and potential blocking agents (a) and to discover and assess the clinical potential of autoantibody signatures for diagnosis of colorectal cancer and Alzheimer’s disease (b). (a) Reprinted with permission from ref 80. Copyright 2022 Elsevier. (b) Created with biorender.com based on ref 32.

Recently, electrochemical biotechnologies have emerged as effective tools not only for the determination of biomarkers, but also for their discovery and assessment of their clinical potential. By leveraging these electrochemical technologies, the diagnostic value of autoantibody signatures and various isotypes targeting autoantigens of different origins have been discovered and comprehensively evaluated. This progress has significantly advanced early-stage diagnosis, monitoring and prognosis of chronic diseases, including colorectal cancer, autoimmune disorders and Alzheimer’s disease (Figure 6b).^32,73,81^

In the context of infectious diseases, multianalyte technologies have been effectively used to monitor viral loads by analyzing specific genetic material while assessing the immune and inflammatory responses of infected individuals,^75^ providing a global view of the infection status. The performance of these technologies has also been exploited to measure, at the same time as viral load, the levels of antibiotics used as preventive measures against bacterial co-infections or superinfections, which often arise due to immune system impairment caused by viral infections.^77^ This dual monitoring ensures appropriate therapeutic interventions and helps to manage possible complications arising from secondary bacterial infections.

### Shaping New Scenarios and Applications

The versatility in the design, integration and application of electrochemical biotools has allowed an ever-increasing range of possibilities for their use. These technologies have successfully ventured into increasingly challenging areas that were previously underexplored. Beyond their established role in clinical diagnosis and prognosis, significant advances in recent years have highlighted the broad appeal of electroanalytical technologies to answer a wide variety of challenging questions of relevance to health today, including the following:

- Assessing the predisposition and/or severity with which certain viral infections are experienced by determining the serum level of metabolic markers, such as fatty acids derived from dietary intake.^82^
- Interrogating the mutational status (homo/heterozygous) of genes relevant to oncology therapies.^46,47^ These technologies hold great promise for personalized medicine, not only in cancer but also in other diseases, such as Alzheimer’s disease, where *ApoE4* homozygosity has recently been identified as a novel genetic form of the disease.^83^
- Detecting minimal residual disease by analyzing specific markers of protein^84^ or genetic nature (such as fusion transcripts and ctDNA).^85,86^

- Assisting in personalized therapy by determining the pharmacokinetics of pharmaceuticals (e.g., assessing individual ability to absorb widely used chemotherapeutics, such as 5-fluorouracil, by monitoring their serum levels in perfused oncology patients)^87^ and biopharmaceuticals (e.g., therapeutic monoclonal antibodies or their fragments).^88^
- Shedding light on the role of the methylome at both DNA and RNA levels in key oncological scenarios.^70,89^

- Advancing the understanding of serum autoantibodies in diseases such as cancer and Alzheimer’s disease by ingeniously combining the opportunities afforded by cutting-edge proteomics for marker identification with technologies that produce nature-inspired receptors.^32^
- Quantifying the processes of natural and/or acquired immunity against viral infections, and implementing personalized strategies for diagnosis, follow-up and vaccination with selectivity towards specific variants.^31^
- Contributing to the improved management of diabetes through precise simultaneous, minimally invasive and decentralized monitoring of key marker pairs such as insulin and glucose;^68^ insulin and cortisol;^67^ and insulin and glucagon.^34,69^

- Determining biomarkers in exhaled breath to usher in a new era of promising biomedical technology, offering advantages such as portability, painlessness, cost-effectiveness, and ease of use (Figure 7a).^90^
- *In situ* and real-time tracking of signaling molecules or toxins in plants (Figure 7b)^91–95^ and soils^96^ offering valuable insights to support the sustained precision health of agriculture and the environment.

**Figure 7:**
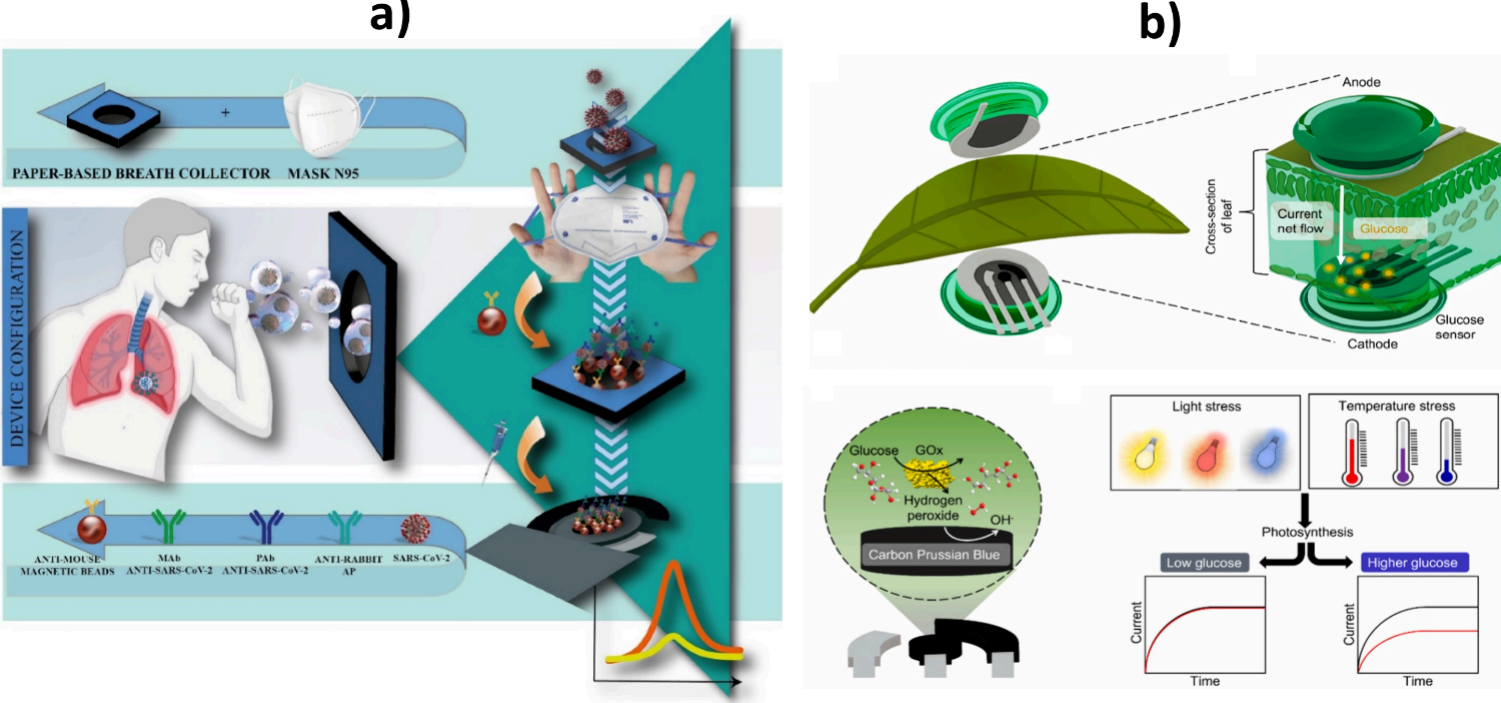
Shaping new scenarios with electroanalytical technologies. Electroanalytical technologies developed to detect SARS-CoV-2 in exhaled breath (a) and for real-time, *in situ* and *in vivo* approaches for glucose monitoring in plants (b). (a) Reprinted with permission from ref 90. Copyright 2024 ACS. (b) Reprinted with permission from ref 91. Copyright 2023 Elsevier.

### Fully Integrated Electrochemical Biosensing Technologies

Despite the success of glucose biosensors, with about 85% of the market, commercial translation of electrochemical biosensing technologies for other biomarkers remains slow. A key challenge is to identify technologies with sufficiently large commercial potential (comparable to glucose) to justify the high development and regulatory costs of new POC diagnostic devices. In addition, technical barriers in translating these biosensing devices from the laboratory to the market extend development timelines and increase costs, making investment in diagnostic technologies riskier even when there is significant market demand. To address these challenges, great efforts have been invested in the development of fully integrated and decentralized electrochemical biosensing technologies.^26,97,98^ Progress in this field is underpinned by advances in several key areas: microfabrication and microelectronics, new bioreceptors that enable single-pot, single-step, reagent-free and/or near real-time response assays, and multifunctional structures that enable *all-in-one* biosensors fabrication without compromising their performance.^99^ In addition, AI and machine learning (ML) are being leveraged to improve data processing, enabling the development of smart efficient, safe, reliable, and ethically sound healthcare systems. Another very promising avenue is the integration of multimode/signal biosensors, which use multiple sensing modalities to improve accuracy, sensitivity and selectivity. This approach not only improves throughput, but also expands analyte coverage and increases versatility, providing synergistic effects for next-generation diagnostic technologies.^100^

Examples of fully integrated electrochemical biodetection technologies include an *all-in-one* electrochemical immunodetection platform for the detection of salivary cotinine,^101^ a centrifugal microfluidic chip involving voltammetric and sandwich aptamer-based detection of vascular endothelial growth factor 165 (VEGF165) in whole blood,^102^ and a wearable integrated aptasensor for rapid electrochemical detection of multiple drugs in sweat^103^ (Figure 8).

**Figure 8:**
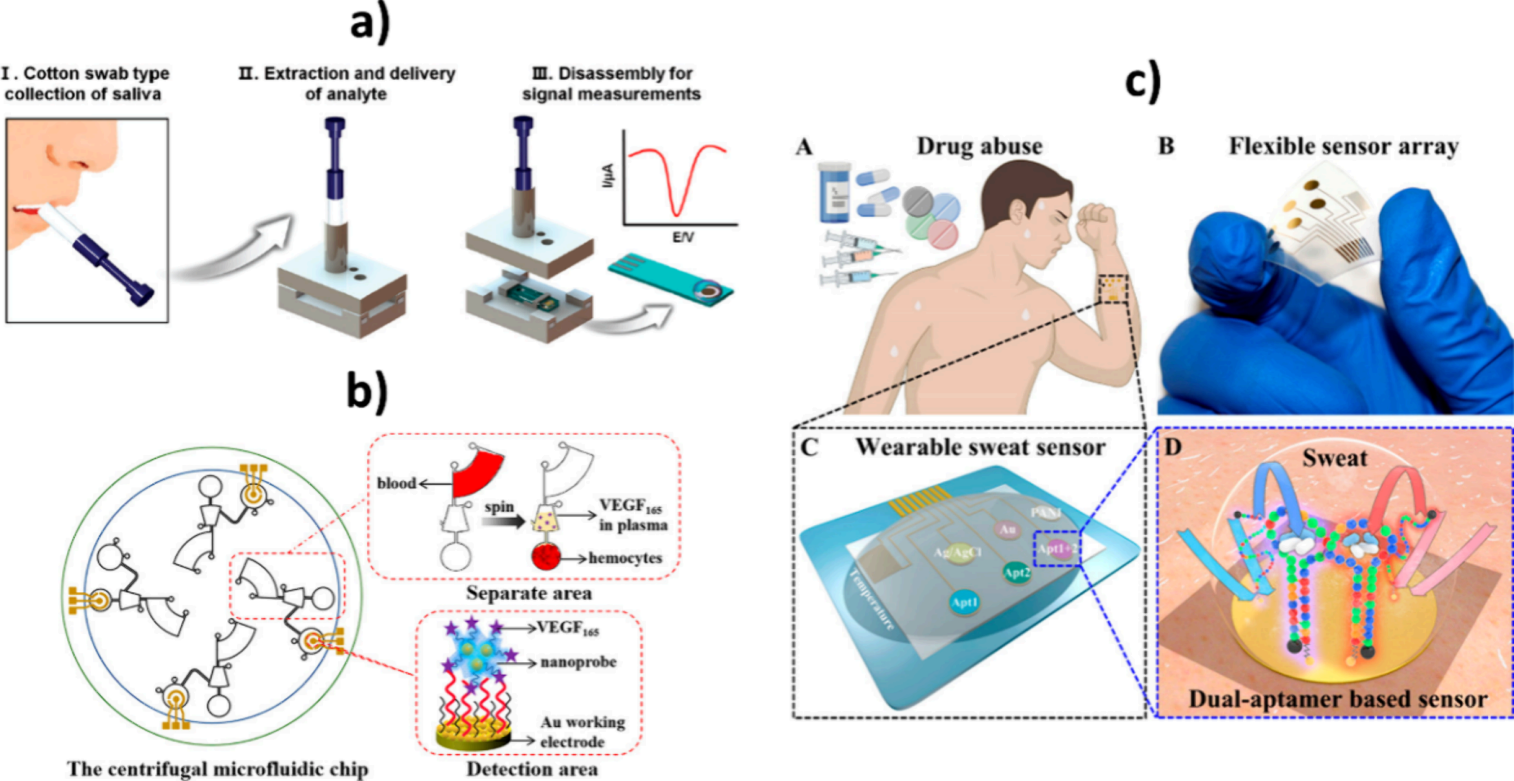
Integrated electrochemical biodetection technologies for tracking cotinine in saliva (a), VEGF165 in whole blood (b), and CRP in sweat (c). (a) Reprinted with permission from ref 101. Copyright 2020 RSC. (b) Reprinted with permission from ref 102. Copyright 2022 ACS. (c) Reprinted with permission from ref 103. Copyright 2023 NPG.

## Bioelectroanalytical Technologies: Today and Tomorrow

Advances in electroanalytical technologies, driven and supported by innovations in other areas, have proven to be invaluable for their versatility in determining biomarkers at multiple omics levels. These technologies now allow us to gain insight into the possible predispositions to suffer certain diseases and disorders by monitoring genomic markers, to understand the dynamic processes that affect individuals through the evaluation of transcriptomic markers, and to assess current health status by means of proteomic and metabolomic markers. They also provide significant opportunities and advantages in nutrition and personalized therapies, by personalizing dietary recommendations to optimize health based on genetic, phenotypic and dietary information, and by tracking minimal residual disease and monitoring (bio)­drugs for personalized treatment strategies, respectively.

In this dynamic and thriving ecosystem, with numerous open fronts and infinite possibilities imagined collectively that fortunately are gradually becoming a reality, an exciting and challenging future lies ahead for both the scientific community and society. A horizon is envisioned that will keep researchers motivated and excited, to make (bio)­electroanalytical technologies available to society to explore new biological matrices (such as nasal exudates, cerebrospinal fluid and feces) and to identify and valorize biomarkers that remain undiscovered or undervalued to date.

Recent research underscores the growing interest and opportunity to leverage these technologies for the detection of less explored but very promising markers. Multifunctional markers contemplated in the roadmap, which may serve as diagnostic indicators, markers of minimal residual disease, and potential therapeutic targets, include nucleosomes, neutrophil extracellular traps, and hidden vulnerabilities within the genome, such as non-canonical structures like G-quadruplex and i-motifs, and fusion genes. In addition, the transcriptome (including transcript fusions and noncoding RNAs) and proteome (encompassing the dark, phantom and neoproteomes) provide valuable information that is increasingly becoming the focus of innovative diagnostic and therapeutic approaches.

Current efforts are focused on advancing autoantibody research, with the goal of improving their valorization using these enabling technologies for early diagnosis, monitoring, and prognosis of chronic diseases. Furthermore, these efforts seek to identify new markers and therapeutic targets, such as neoantigens (primarily tumor-specific antigens that are only expressed in tumor cells), to guide more efficient therapeutic interventions.^104^ Achieving this goal will require continued innovation in the design, production, and integration of novel receptors into these technologies, specifically, proteoforms derived from alternative splicing and proteins expressed in mammalian cells. Unlike those produced through bacterial or cell-free expression systems, the mammalian cell-derived proteins replicate more faithfully their native human forms, including folding and post-translational modifications. This leads to preparations that, in addition to ensuring protein functionality, stability and solubility, are more homogeneous and provide more consistent, accurate and reliable results in clinical applications.

In terms of precision nutrition, advances in electroanalytical technologies provide great promise for determining the metabolites produced after food ingestion, particularly those that are involved in altered metabolic pathways. This includes the assessment of oxidative DNA damage and toxic molecules produced during food cooking, such as advanced glycation end products, chloropropanols, glycidol and their fatty acid esters. Furthermore, the detection of nucleic acids in food, which may contribute to mutations linked to cancer and other diseases, is of particular interest. These technologies will open invaluable insights into the intricate connection between diet, metabolism and health, paving the way for more personalized and precise nutritional interventions.

It is indisputable that the performance of electrochemical (bio)­tools will face increasing challenges, particularly in terms of simplicity (with the aim of developing “one-step” and “one-pot” technologies), along with sensitivity, robustness, reusability, and antifouling capabilities. The use of nanoporous substrates,^105^ innovative (bio)­receptors (such as plantibodies, nanobodies, functional antibodies, bispecific antibodies, recombinant antibody fragments, antipeptide antibodies, and multi-antigen peptides with immunoglobulin affinity), more stable and robust chemical (bio)­functionalization strategies, and new developments in isothermal nucleic acid amplification technologies will contribute to overcoming these challenges.

Moreover, the advent of gene editing and AI tools is set to revolutionize these biotechnologies. Indeed, the synergy between AI and electrochemical biotechnologies promises a new era in their design, functionality and performance, despite the ethical and standardization concerns that remain around AI in patient-centered research. This collaboration will improve accuracy, efficiency, accessibility, scalability, reliability, and speed in clinical and real-world applications. The potential to develop sustainable POC devices, including for self-diagnosis or home use, is very promising. These devices may deliver real-time early detection and improved patient outcomes, marking a transformative advancement in personalized healthcare technology.^51,106,107^ On the positive side, the COVID-19 pandemic has awakened the entire scientific community to the urgency of creating commercialization-ready platforms and has raised the bar for bioanalytical assay requirements used for POC diagnostics, which is undoubtedly accelerating the development cycle of potentially translatable and marketable biosensing technologies.^26^

Besides prioritizing the development of multiplexed and/or multi-omics biosensing tools, emphasis will be placed on improving their versatility in design and implementation. This versatility will be exploited to create multi-purpose technologies that tackle closely related personalized medicine and nutrition needs, thus contributing to precision health. Some promising examples currently on the horizon include:

- Protein allergens and immunoglobulins: Design (bio)­electrochemical tools to detect simultaneously protein allergens in foods and serum levels of specific immunoglobulin isotypes associated with the diagnosis and prognosis of allergies or intolerances (IgEs and IgG4s).
- Fatty acids and viral infections: Develop electroanalytical technologies to quantify the level of fatty acids, such as arachidonic acid, that are linked to viral infection trends and risks. These tools may be applied to both serum and food matrices to help identify risks and prevent viral infections.
- Genetic risk and metabolic pathways: Develop biotools to identify biomarkers involved in altered metabolic pathways in diseases and in the genetic predisposition to develop them (e.g. homozygosity in *ApoE4* for Alzheimer’s disease) with the objective of prevention.^108^

Integration of these technologies into healthcare will enable more accurate diagnostics and effective monitoring, improving health outcomes. The future of medicine will be more precise, with a clear shift towards precision approaches, not only in healthcare, but also in precision agriculture, which optimizes the use of resources in a more sustainable way. In a context where climate, food security and health are interconnected, the development of advanced technologies for accurate information will be key to building resilient and sustainable health systems.

However, it cannot be overlooked that the translation of these electroanalytical technologies into clinical practice must face several critical challenges. The lack of standardization in sensor fabrication and functionalization can be a source of variability in the performance and reproducibility of these technologies in different settings. Although work continues relentlessly to improve their performance to allow direct analysis in complex biological matrices, such as whole blood or saliva, with significant biofouling or interfering components, laborious sample pretreatment is often still required, making workflow integration difficult. Although it is indisputable that both the integration of nanomaterials and the rational design of surfaces have significantly improved the sensitivity and detectability of these biotechnologies, they also increase complexity and manufacturing costs, which limits their large-scale production and makes regulatory compliance difficult.

From an economic standpoint, the high cost of some substrates or their required processing in clean rooms, and integration with digital health infrastructure pose barriers to commercialization and widespread adoption, especially in low-resource settings. In addition, electrochemical biotechnologies must also prove to be cost-effective relative to established methods, which benefit from economies of scale and existing infrastructures.

From an ethical point of view, the proliferation of designs connected to smartphones or cloud-based healthcare platforms raises significant concerns in terms of data privacy and user ownership and consent, particularly when employed outside conventional healthcare systems. In addition, continuous or passive monitoring via wearable electrochemical devices also challenges user autonomy and puts surveillance systems on alert. On the other hand, unequal access to these devices and the type of diagnosis they offer could further increase existing health inequalities among the population. Moreover, the translation of devices used in academic research into commercially viable prototypes is another hurdle to be overcome, requiring the joint involvement of researchers and engineers to solve the complex challenges of biosensor hardware development, together with companies with access to the technology of manufactory sensors and their modification in an efficient and controllable manner.^109^ In addition, a major obstacle is the stringent regulatory framework governing clinical diagnostics, which requires extensive validation, standardization and compliance with guidelines such as those imposed by the FDA or EMA. These processes can be time-consuming and financially burdensome, especially for companies that also face challenges related to intellectual property protection, technology transfer and complex commercialization pathways. Finally, premature reliance of electroanalytical biotechnologies results without adequate clinical validation may lead to errors and inappropriate medical decisions. Thus, despite the unique and very promising advantages offered by bioelectroanalytical technologies in patient care and personalized medicine, coordinated and efficient efforts are required to overcome the significant regulatory, legal and economic barriers arising for their sustainable translation to the patient’s bedside. All of this helps to understand why all the technologies highlighted in this perspective article are in Technological Readiness Levels (TLRs) 1–3.

Despite all these challenges, the transformation that we are already witnessing thanks to these enabling technologies will also empower people, encouraging self-care and more efficient use of resources. Even though the road ahead maybe be bumpy, real-time data analytics envisions a bright future. Health technologies will continuously evolve, enabling better healthcare management and new possibilities.

Furthermore, as this revolution progresses, human health, but also animal and plant health will improve, with the resulting transformation of precision health in all walks of life. Electrochemical biotechnologies are envisioned as accessible and adaptable scientific tools with the potential to improve clinical outcomes, foster personalized treatments, push the boundaries of precision healthcare and shape a healthier and more resilient future for society.

These technologies, geared toward minimally invasive diagnostics and sustainable practices, will lead us to a future of democratized precision healthcare that embraces both humanity and all living systems on Earth. And all this, yet another proof of the potential of science and research to improve our lives.

## References

[1] What will it take to make precision health a global reality. Nat. Med. 2024, 30, 1793–1794. 10.1038/s41591-024-03163-8. 39030406

[2] Roberts M. C., Holt K. E., Del Fiol G., Baccarelli A. A., Allen C.G. (2024). Precision public health in the era
of genomics and
big data. Nat. Med..

[3] Roman S., Campos-Medina L., Leal-Mercado L. (2024). Personalized nutrition: the end of
the one-diet-fits-all era. Front. Nutr..

[4] Campuzano S., Barderas R., Yáñez-Sedeño P., Pingarrón J. M. (2021). Electrochemical biosensing to assist multiomics analysis
in precision medicine. Curr. Op. Electrochem..

[5] Campuzano S., Pingarrón J. M. (2023). Electrochemical
affinity biosensors: Pervasive devices
with exciting alliances and horizons ahead. ACS Sens..

[6] Campuzano S., Barderas R., Moreno-Casbas M. T., Almeida Á., Pingarrón J. M. (2024). Pursuing
precision in medicine and nutrition: the rise of electrochemical biosensing
at the molecular level. Anal. Bioanal. Chem..

[7] Kalita N., Gogoi S., Minteer S. D., Goswami P. (2023). Advances in bioelectrode
design for developing electrochemical biosensors. ACS Meas. Sci. Au..

[8] Sankar K., Kuzmanović U., Schaus S. E., Galagan J. E., Grinstaff M. W. (2024). Strategy,
design, and fabrication of electrochemical biosensors: A tutorial. ACS Sens..

[9] Dincer C., Bruch R., Costa-Rama E., Fernández-Abedul M. T., Merkoçi A., Manz A., Urban G. A., Güder F. (2019). Disposable
sensors in diagnostics, food, and environmental monitoring. Adv. Mater..

[10] Barros
Azeredo N. F., Ferreira Santos M. S., Sempionatto J. R., Wang J., Angnes L. (2022). Screen-printed technologies combined
with flow analysis techniques: Moving from benchtop to everywhere. Anal. Chem..

[11] Arduini F. (2022). Electrochemical
paper-based devices: When the simple replacement of the support to
print ecodesigned electrodes radically improves the features of the
electrochemical devices. Curr. Op. Electrochem..

[12] Marchianò V., Tricase A., Cimino A., Cassano B., Catacchio M., Macchia E., Torsi L., Bollella P. (2025). Inside out: Exploring
edible biocatalytic biosensors for health monitoring. Bioelectrochemistry.

[13] Huang Y., Yan T., Wu M., Li X., Gao X., Yang Y., He N., Song J., Cui Y., Chen H., Xu L.-P. (2024). Bioinspired
superwettable electrode towards sensitive detection of myocardial
infarction-specific miRNA. Sens. Actuators B:
Chem..

[14] Zhao Y., Jin K. Q., Li J. D., Sheng K. K., Huang W. H., Liu Y. L. (2023). Flexible and stretchable
electrochemical sensors for
biological monitoring. Adv. Mater..

[15] Yin L., Lv J., Wang J. (2020). Structural
innovations in printed, flexible, and stretchable
electronics. Adv. Mater. Technol..

[16] Brasier N., Wang J., Gao W., Sempionatto J. R., Dincer C., Ates H. C., Güder F., Olenik S., Schauwecker I., Schaffarczyk D., Vayena E., Ritz N., Weisser M., Mtenga S., Ghaffari R., Rogers J. A., Goldhahn J. (2024). Applied body-fluid
analysis by wearable devices. Nature.

[17] Zhou J., Zhou S., Fan P., Li X., Ying Y., Ping J., Pan Y. (2024). Implantable electrochemical microsensors
for in vivo monitoring of animal physiological information. Nanomicro. Lett..

[18] Abdullah H., Phairatana T., Jeerapan I. (2022). Tackling the challenges
of developing
microneedle-based electrochemical sensors. Microchim.
Acta.

[19] Kim G., Ahn H., Chaj Ulloa J., Gao W. (2024). Microneedle sensors for dermal interstitial
fluid analysis. Med-X..

[20] Ye C., Lukas H., Wang M., Lee Y., Gao W. (2024). Nucleic acid-based
wearable and implantable electrochemical sensors. Chem. Soc. Rev..

[21] Rabiee N., Rabiee M. (2024). Wearable aptasensors. Anal. Chem..

[22] Gooding J. J. (2025). Some of
our favorite papers from the first 10 years of *ACS Sensors*. ACS Sens..

[23] Gutiérrez-Capitán M., Sanchís A., Carvalho E. O., Baldi A., Vilaplana L., Cardoso V.F., Calleja Á., Wei M., de la Rica R., Hoyo J., Bassegoda A., Tzanov T., Marco M. P., Lanceros-Méndez S., Fernández-Sánchez C. (2023). Engineering
a point-of-care paper-microfluidic electrochemical device applied
to the multiplexed quantitative detection of biomarkers in sputum. ACS Sens..

[24] Nandhakumar P., Muñoz San Martin C., Arevalo B., Ding S., Lunker M., Vargas E., Djassemi O., Campuzano S., Wang J. (2023). Redox cycling amplified
electrochemical lateral-flow immunoassay:
Toward decentralized sensitive insulin detection. ACS Sens..

[25] Schellberg B. G., Koppes R. A., Koppes A. N. (2025). Recent advances in integrated organ-chip
sensing toward robust and user-friendly systems. J. Biomed. Mater. Res., Part A.

[26] Akhlaghi A. A., Kaur H., Adhikari B. R., Soleymani L. (2024). Editors’
Choice—Challenges and opportunities for developing electrochemical
biosensors with commercialization potential in the point-of-care diagnostics
market. ECS Sensors Plus.

[27] Montero-Calle A., Aranguren-Abeigon I., Garranzo-Asensio M., Guzman-Aranguez A., Povés C., Fernández-Aceñero M. J., Martínez-Useros J., Sanz R., Dziaková J., Rodríguez-Cobos J., Solís-Fernández G., Povedano E., Gamella M., Torrente-Rodríguez R. M., Alonso-Navarro M., de los Ríos V., Casal J. I., Domínguez G., Pingarrón J. M., Peláez-García A., Campuzano S., Barderas R. (2021). Multiplexed biosensing diagnostic
platforms detecting autoantibodies to tumor-associated antigens from
exosomes released by CRC cells and tissue samples showed high diagnostic
ability for colorectal cancer. Engineering.

[28] Campuzano S., Pedrero M., Barderas R., Pingarrón J. M. (2024). Breaking
barriers in electrochemical biosensing using bioinspired peptide and
phage probes. Anal. Bioanal. Chem..

[29] Valverde A., Montero-Calle A., Arévalo B., San Segundo-Acosta P., Serafín V., Alonso-Navarro M., Solís-Fernández G., Pingarrón J. M., Campuzano S., Barderas R. (2021). Phage-derived and aberrant
Halotag peptides immobilized on magnetic microbeads for amperometric
biosensing of serum autoantibodies and Alzheimer’s disease
diagnosis. Anal. Sens..

[30] Montero-Calle A., Garranzo-Asensio M., Torrente-Rodríguez R. M., Ruiz-Valdepeñas
Montiel V., Poves C., Dziaková J., Sanz R., Díaz Del Arco C., Pingarrón J. M., Fernández-Aceñero M. J., Campuzano S., Barderas R. (2023). p53 and p63 proteoforms derived from alternative splicing
possess differential seroreactivity in colorectal cancer with distinct
diagnostic ability from the canonical proteins. Cancers (Basel).

[31] Torrente-Rodríguez R. M., Montero-Calle A., San Bartolomé C., Cano O., Vazquez M., Iglesias-Caballero M., Corral-Lugo A., McConnell M. J., Pascal M., Mas V., Pingarrón J. M., Barderas R., Campuzano S. (2022). Towards control and oversight of
SARS-CoV-2 diagnosis and monitoring through multiplexed quantitative
electroanalytical immune response biosensors. Angew. Chem. Int. Ed..

[32] Povedano E., Garranzo-Asensio M., Montero-Calle A., Valverde A., Dalmasso P., San Segundo-Acosta P., Cano O., Vázquez M., Mas V., Fernández-Aceñero M. J., Rivas G., Pingarrón J. M., Campuzano S., Barderas R. (2025). Novel 6xHis/HaloTag mammalian expressed
autoantigens for the detection of humoral response with multiplexed
electrochemical biosensors: A breakthrough in colorectal cancer and
Alzheimer’s disease personalized diagnostics. Biosens. Bioelectron..

[33] Vargas E., Zhang F., Ben Hassine A., Ruiz-Valdepeñas
Montiel V., Mundaca-Uribe R., Nandhakumar P., He P., Guo Z., Zhou Z., Fang RH., Gao W., Zhang L., Wang J. (2022). Using cell membranes as recognition
layers to construct ultrasensitive and selective bioelectronic affinity
sensor. J. Am. Chem. Soc..

[34] Nandhakumar P., Sun L., Li Z., Cheung C., Nguyen L., Ding S., Gao W., Zhang L., Wang J. (2024). Biomimetic cell membrane layers for
the detection of insulin and glucagon. Anal.
Chem..

[35] Parolo C., Idili A., Heikenfeld J., Plaxco K. W. (2023). Conformational-switch
biosensors as novel tools to support continuous, real-time molecular
monitoring in lab-on-a-chip devices. Lab Chip.

[36] Silva S. M., Li M., Mendes A. X., Moulton S. E. (2023). Reagentless protein-based electrochemical
biosensors. Analyst.

[37] Arévalo B., Blázquez-García M., Valverde A., Serafín V., Montero-Calle A., Solís-Fernández G., Barderas R., Campuzano S., Yáñez-Sedeño P., Pingarrón J. M. (2022). Binary
MoS_2_ nanostructures as nanocarriers
for amplification in multiplexed electrochemical immunosensing: simultaneous
determination of B cell activation factor and proliferation-induced
signal immunity-related cytokines. Microchim.
Acta.

[38] Arévalo B., Blázquez-García M., Valverde A., Serafín V., Yáñez-Sedeño P., Campuzano S., Pingarrón J. M. (2023). First electrochemical bioplatforms
to determine anti-centromere
B antibodies: critical comparison between integrated and magnetic
bead-assisted strategies using His-tag chemistry. Sens. Diagn..

[39] Garranzo-Asensio M., Guzmán-Aránguez A., Povedano E., Ruiz-Valdepeñas
Montiel V., Poves C., Fernandez-Aceñero M. J., Montero-Calle A., Solís-Fernández G., Fernandez-Diez S., Camps J., Arenas M., Rodriguez-Tomas E., Joven J., Sanchez-Martinez M., Rodriguez N., Dominguez G., Yáñez-Sedeño P., Pingarrón J. M., Campuzano S., Barderas R. (2020). Multiplexed monitoring
of a novel autoantibody diagnostic signature of colorectal cancer
using HaloTag technology-based electrochemical immunosensing platform. Theranostics.

[40] Campuzano S., Pedrero M., Barderas R., Pingarrón J. M. (2022). Empowering
electrochemical biosensing through nanostructured or multifunctional
nucleic acid or peptide biomaterials. Adv. Mater.
Technol..

[41] Razzino C. A., Serafín V., Gamella M., Pedrero M., Montero-Calle A., Barderas R., Calero M., Lobo A. O., Yáñez-Sedeño P., Campuzano S., Pingarrón J. M. (2020). An electrochemical immunosensor using
gold nanoparticles-PAMAM-nanostructured screen-printed carbon electrodes
for tau protein determination in plasma and brain tissues from Alzheimer
patients. Biosens. Bioelectron..

[42] Soto D., Serafín V., Pedrero M., Pingarrón J. M., Campuzano S., Orozco J. (2024). Hierarchical Au@Pt nanoparticle/amino
benzoic acid polymer-based hybrid material for labeled and label-free
detection of interleukin-6: a comparative assessment. Microchim. Acta.

[43] Ruiz-Valdepeñas
Montiel V., Povedano E., Vargas E., Torrente-Rodríguez R. M., Pedrero M., Reviejo A. J., Campuzano S., Pingarrón J. M. (2018). Comparison of different strategies for the development
of highly sensitive electrochemical nucleic acid biosensors using
neither nanomaterials nor nucleic acid amplification. ACS Sens..

[44] Hu X., Wei W., Li X., Yang Y., Zhou B. (2024). Recent advances
in
ratiometric electrochemical sensors for food analysis. Food Chem. X..

[45] Moranova L., Stanik M., Hrstka R., Campuzano S., Bartosik M. (2022). Electrochemical LAMP-based assay for detection of RNA
biomarkers in prostate cancer. Talanta.

[46] Sebuyoya R., Valverde A., Moranova L., Strmiskova J., Hrstka R., Montiel V. R.-V., Pingarron J. M., Barderas R., Campuzano S., Bartosik M. (2023). Dual detection system
for cancer-associated point mutations assisted by a multiplexed LNA-based
amperometric bioplatform coupled with rolling circle amplification. Sens. Actuators B-Chem..

[47] Sebuyoya R., Sevcikova S., Yusuf B., Bartosik M. (2025). Integrating
isothermal
amplification techniques and LNA-based AI-assisted electrochemical
bioassay for analysis of KRAS G12V point mutation. Talanta.

[48] Zou S., Li J., Lu S., Li D., Liu Y., Zhang W., Cui X., Gooding J.J., Tian K., Liu G. (2024). CRISPR-Cas12a immunosensing
on glass fiber for point-of-care quantification of multiple inflammation
biomarkers in osteoarthritis. Device.

[49] Wachholz D., Kubota L. T. (2024). CRISPR-based
electrochemical biosensors:
an alternative for point-of-care diagnostics?. Talanta.

[50] Giordano G. F., Ferreira L. F., Bezerra Í. R.
S., Barbosa J. A., Costa J. N. Y., Pimentel G. J. C., Lima R. S. (2023). Machine learning
toward high-performance electrochemical sensors. Anal. Bioanal. Chem..

[51] Bhaiyya M., Panigrahi D., Rewatkar P., Haick H. (2024). Role of machine
learning
assisted biosensors in point-of-care-testing for clinical decisions. ACS Sens..

[52] Sempionatto J. R., Khorshed A. A., Ahmed A., De Loyola e Silva A. N., Barfidokht A., Yin L., Goud K. Y., Mohamed M. A., Bailey E., May J., Aebischer C., Chatelle C., Wang J. (2020). Epidermal enzymatic biosensors for
sweat vitamin c: toward personalized nutrition. ACS Sens..

[53] Ruiz-Valdepeñas
Montiel V., Sempionatto J. R., Vargas E., Bailey E., May J., Bulbarello A., Dusterloh A., Matusheski N., Wang J. (2021). Decentralized vitamin C & D dual biosensor chip: Toward personalized
immune system support. Biosens. Bioelectron..

[54] Fortunati S., Giannetto M., Giliberti C., Mattarozzi M., Bertucci A., Careri M. (2024). Magnetic beads
as versatile tools
for electrochemical biosensing platforms in point-of-care testing. Anal. Sens..

[55] Wang J. (2016). Self-propelled
affinity biosensors: Moving the receptor around the sample. Biosens. Bioelectron..

[56] Gallo-Orive Á., Moreno-Guzmán M., Sanchez-Paniagua M., Montero-Calle A., Barderas R., Escarpa A. (2024). Gold nanoparticle-decorated
catalytic micromotor-based aptassay for rapid electrochemical label-free
amyloid-β42 oligomer determination in clinical samples from
Alzheimer’s patients. Anal. Chem..

[57] Gordón
Pidal J. M., Moreno-Guzmán M., Montero-Calle A., Valverde A., Pingarrón J. M., Campuzano S., Calero M., Barderas R., López M. Á., Escarpa A. (2024). Micromotor-based electrochemical immunoassays for reliable
determination of amyloid-β (1–42) in Alzheimer’s
diagnosed clinical sample. Biosens. Bioelectron..

[58] Lazanas A. Ch., Prodromidis M. I. (2023). Electrochemical
impedance spectroscopy–A Tutorial. ACS
Meas. Sci. Au.

[59] Gutiérrez-Gálvez L., Del Caño R., Menéndez-Luque I., García-Nieto D., Rodríguez-Peña M., Luna M., Pineda T., Pariente F., García-Mendiola T., Lorenzo E. (2022). Electrochemiluminescent
nanostructured DNA biosensor for SARS-CoV-2 detection. Talanta.

[60] Campuzano S., Gamella M., Pedrero M., Pingarrón J. M. (2023). Affinity
bioelectroanalysis in cellular-level biomarker driven modern precision
cancer diagnosis. TrAC, Trends Anal. Chem..

[61] Sumitha M. S., Xavier T. S. (2023). Recent advances
in electrochemical biosensors –
A brief review. Hybrid Advances.

[62] Li S., Zhang H., Zhu M., Kuang Z., Li X., Xu F., Miao S., Zhang Z., Lou X., Li H., Xia F. (2023). Electrochemical
biosensors for whole blood analysis: recent progress,
challenges, and future perspectives. Chem. Rev..

[63] Kim J., Jeong J., Ko S. H. (2024). Electrochemical biosensors for point-of-care
testing. Bio-Des Manuf..

[64] Cao Y., Xia J., Li L., Zeng Y., Zhao J., Li G. (2024). Electrochemical
biosensors for cancer diagnosis: multitarget analysis to present molecular
characteristics of tumor heterogeneity. JACS
Au.

[65] Klebes A., Ates H. C., Verboket R. D., Urban G. A., von Stetten F., Dincer C., Fruh S. M. (2024). Emerging multianalyte biosensors
for the simultaneous detection of protein and nucleic acid biomarkers. Biosens. Bioelectron..

[66] Sharafeldin M., Rusling J. F. (2023). Multiplexed electrochemical assays
for clinical applications. Curr. Opin. Electrochem..

[67] Vargas E., Povedano E., Krishnan S., Teymourian H., Tehrani F., Campuzano S., Dassau E., Wang J. (2020). Simultaneous
cortisol/insulin microchip detection using dual enzyme tagging. Biosens. Bioelectron..

[68] Vargas E., Teymourian H., Tehrani F., Eksin E., Sánchez-Tirado E., Warren P., Erdem A., Dassau E., Wang J. (2019). Enzymatic/immunoassay
dual-biomarker sensing chip: towards decentralized insulin/glucose
detection. Angew. Chem. Int. Ed..

[69] Nandhakumar P., Djassemi O., Raucci A., Chang A. Y., Cheung C., Dugas Y., Silberman J., Morales-Fermin S., Sandhu S. S., Reynoso M., Saha T., Cinti S., Wang J. (2024). Simultaneous and rapid detection of glucose and insulin: coupling
enzymatic and aptamer-based assays. Anal. Chem..

[70] Povedano E., Pérez-Ginés V., Torrente-Rodríguez R. M., Rejas-González R., Montero-Calle A., Peláez-García A., Feliú J., Pedrero M., Pingarrón J. M., Barderas R., Campuzano S. (2025). Tracking globally
5-methylcytosine and its oxidized derivatives in colorectal cancer
epigenome using bioelectroanalytical technologies. ACS Sens..

[71] Shanmugam S. T., Steijlen A., Laurijssen D., Campos R., Steckel J., Daems W., Bassini S., Daems E., De Wael K. (2024). A 96-Well
LED array for multiplexed photoelectrochemical detection of nucleic
acids. Anal. Chem..

[72] Povedano E., Gamella M., Torrente-Rodríguez RM., Ruiz-Valdepeñas
Montiel V., Montero-Calle A., Solís-Fernández G., Navarro-Villoslada F., Pedrero M., Peláez-García A., Mendiola M., Hardisson D., Feliú J., Barderas R., Pingarrón J. M., Campuzano S. (2021). Multiplexed
magnetic beads-assisted amperometric bioplatforms for global detection
of methylations in nucleic acids. Anal. Chim.
Acta..

[73] Arévalo B., Serafín V., Garranzo-Asensio M., Montero-Calle A., Barderas R., Yáñez-Sedeño P., Campuzano S., Pingarrón J. M. (2023). Anti-double stranded DNA antibodies:
electrochemical isotyping in autoimmune and neurological diseases. Anal. Chim. Acta.

[74] Genco E., Modena F., Sarcina L., Björkström K., Brunetti C., Caironi M., Caputo M., Demartis V. M., Di Franco C., Frusconi G., Haeberle L., Larizza P., Mancini M. T., Österbacka R., Reeves W., Scamarcio G., Scandurra C., Wheeler M., Cantatore E., Esposito I., Macchia E., Torricelli F., Viola F. A., Torsi L. A. (2023). single-molecule bioelectronic portable
array for early diagnosis of pancreatic cancer precursor. Adv. Mater..

[75] Torrente-Rodriguez R.
M., Lukas H., Tu J., Min J., Yang Y., Xu C., Rossiter H. B., Gao W. (2020). SARS-CoV-2 RapidPlex: A graphene-based
multiplexed telemedicine platform for rapid and low-cost covid-19
diagnosis and monitoring. Matter..

[76] Najjar D., Rainbow J., Sharma Timilsina S., Jolly P., de Puig H., Yafia M., Durr N., Sallum H., Alter G., Li J. Z., Yu X. G., Walt D. R., Paradiso J. A., Estrela P., Collins J. J., Ingber D. E. (2022). A lab-on-a-chip
for the concurrent electrochemical detection of SARS-CoV-2 RNA and
anti-SARS-CoV-2 antibodies in saliva and plasma. Nat. Biomed. Eng..

[77] Johnston M., Ates H. C., Glatz R. T., Mohsenin H., Schmachtenberg R., Goppert N., Huzly D., Urban G. A., Weber W., Dincer C. (2022). Multiplexed biosensor for point-of-care
COVID-19 monitoring:
CRISPR-powered unamplified RNA diagnostics and protein-based therapeutic
drug management. Mater. Today..

[78] Torrente-Rodríguez R. M., Campuzano C., Ruiz-Valdepeñas Montiel V., Gamella M., Pingarrón J. M. (2016). Electrochemical
bioplatforms for
the simultaneous determination of interleukin (IL)-8 mRNA and IL-8
protein oral cancer biomarkers in raw saliva. Biosens. Bioelectron..

[79] Gong H., Bao C., Guo X., Tian F., Qiu L., Yang W. (2024). A universal
electrochemical sensor for detection of nucleic acids and protein
based on host-guest recognition of *β*-cyclodextrin
polymer. Microchem. J..

[80] Zouari M., Aissaoui-Zid D., Campuzano S., Barderas R., Srairi-Abid N., Pingarrón J. M., Raouafi N. (2022). Multipurpose E-bioplatform targeting
Kv channels in whole cancer cells and evaluating of their potential
therapeutic. Anal. Chim. Acta..

[81] Arévalo B., Serafín V., Garranzo-Asensio M., Barderas R., Yáñez-Sedeño P., Campuzano S., Pingarrón J. M. (2023). Early and differential autoimmune
diseases diagnosis by interrogating specific autoantibody signatures
with multiplexed electrochemical bioplatforms. Biosens. Bioelectron. X..

[82] Torrente-Rodríguez R. M., Ruiz-Valdepeñas
Montiel V., Iftimie S., Montero-Calle A., Pingarrón J. M., Castro A., Camps J., Barderas R., Campuzano S., Joven J. (2024). Contributing to the management of
viral infections through simple immunosensing of the arachidonic acid
serum level. Microchim. Acta.

[83] Xu Q., Liang Z., Huang Y. (2024). *APOE4* homozygosity
is a new genetic form of Alzheimer’s disease. Nat. Med..

[84] Tejerina-Miranda S., Blázquez-García M., Serafín V., Montero-Calle A., Garranzo-Asensio M., Reviejo A. J., Pedrero M., Pingarrón J. M., Barderas R., Campuzano S. (2023). Electrochemical
biotool for the dual determination of epithelial mucins associated
to prognosis and minimal residual disease in colorectal cancer. Int. J. Biol. Macromol..

[85] Yang L.-Y., Huang J.-L., Lin Y., Cai Q.-Q., Zheng Y.-J., Wu Y., Chen J.-Y., Lin X.-H. (2024). A split-type electrochemical biosensor
using enzyme-linked DNA magnetic beads realizes the detection of BCR/ABL^p210^ fusion gene in clinical samples: Duplex ligation chain
reaction coupled with OR logic gate design. Chem. Eng. J..

[86] Yu L. Y., Chen J. Y., Weng H. J., Lin H. F., Zhang C. J., Yang L. Y., Lin J. Z., Lin X. H., Zhong G. X. (2025). Cell-free
transcription amplification-based split-type electrochemical sensor
using enzyme-linked magnetic microbeads for minimal residual leukemia
detection. Talanta.

[87] Zouari M., Barderas R., Pingarrón J.M., Raouafi N., Campuzano S. (2024). First electrochemical
bioplatform to assist in personalized 5-fluorouracil chemotherapy. Sens. Actuat. B-Chem..

[88] Cunha D. R., Segundo M. A., Quinaz M. B. (2025). Electrochemical
methods for evaluation
of therapeutic monoclonal antibodies: A review. Biosens. Bioelectron..

[89] Li H., Xie X., Liu X., Wu P., He J., Lin F., Shi L., Huang Y. (2024). Ultrasensitive biosensors detecting m^6^A
in blood: achieving early screening and typing of tumors. ACS Sens..

[90] Gutiérrez-Gálvez L., Seddaoui N., Fiore L., Fabiani L., García-Mendiola T., Lorenzo E., Arduini F. (2024). Functionalized N95 face mask with
a chemical-free paper-based collector for exhaled breath analysis:
SARS-CoV-2 detection with a printed immunosensor as a case study. ACS Sens..

[91] Perdomo S. A., De la Paz E., Del Caño R., Seker S., Saha T., Wang J., Jaramillo-Botero A. (2023). Non-invasive in-vivo glucose-based
stress monitoring in plants. Biosens. Bioelectron..

[92] Zhou S., Zhou J., Pan Y., Wu Q., Ping J. (2024). Wearable electrochemical
sensors for plant small-molecule detection. Trends Plant Sci..

[93] Banerji S., Hõrak H., Torop J., Huynh T.-P. (2024). Unravelling
the
secrets of plants: emerging wearable sensors for plants signals and
physiology. Adv. Sensor Res..

[94] Liu W., Zhang Z., Geng X., Tan R., Xu S., Sun L. (2025). Electrochemical sensors for plant
signaling molecules. Biosens. Bioelectron..

[95] Seker S., Surucu O., Economou A., Wang J. (2025). “On-plant”
wearable electrochemical sensor for atmospheric lead monitoring. Talanta.

[96] Pal A., Dubey S. K., Goel S., Kalita P. K. (2024). Portable sensors
in precision agriculture: Assessing advances and challenges in soil
nutrient determination. TrAC, Trends Anal. Chem..

[97] Wu J., Liu H., Chen W., Ma B., Ju H. (2023). Device integration
of electrochemical biosensors. Nat. Rev. Bioeng..

[98] Kalambate P. K., Kumar V., Dhanjai (2025). Decentralized electrochemical biosensors
for biomedical
applications: From lab to home. Next Nanotechnology.

[99] Dong N., Liu S., Li Y., Meng S., Liu Y., Li X., Liu D., You T. (2023). All-in-one fabrication of a ratiometric electrochemical
aptasensor with tetrahedral DNA nanostructure for fumonisin B1 detection. Chem. Commun..

[100] Han Q., Wang H., Wang J. (2024). Multi-mode/signal biosensors: electrochemical
integrated sensing techniques. Adv. Funct. Mater..

[101] Lee K., Yoon T., Yang H.-S., Cha S., Cheon Y.-P., Kashefi-Kheyrabadi L., Jung H.-I. (2020). All-in-one platform
for salivary
cotinine detection integrated with a microfluidic channel and an electrochemical
biosensor. Lab Chip.

[102] He X., Wang X., Ge C., Li S., Wang L., Xu Y. (2022). Detection of vegf_165_ in
whole blood by differential pulse
voltammetry based on a centrifugal microfluidic chip. ACS Sens..

[103] Zhang X., Tang Y., Wu H., Wang Y., Niu L., Li F. (2022). Integrated aptasensor
array for sweat drug analysis. Anal. Chem..

[104] Chakraborty C., Majumder A., Bhattacharya M., Chatterjee S., Lee S.-S. (2024). The landscape of neoantigens and
its clinical applications: From immunobiology to cancer vaccines. CRBIOT.

[105] Zhao C. L., Gao R., Niu Y., Cai B., Zhu Y. (2025). Exploring the diffusion
of DNA strands into nanoporous structures
for establishing a universal electrochemical biosensor. Chem. Sci..

[106] Nashruddin S. N. A. B.
M., Salleh F. H. M., Yunus R. M., Zaman H. B. (2024). Artificial intelligence–powered
electrochemical
sensor: Recent advances, challenges, and prospects. Heliyon.

[107] Cernat A., Groza A., Tertis M., Feier B., Hosu-Stancioiu O., Cristea C. (2024). Where artificial intelligence stands
in the development of electrochemical sensors for healthcare applications-A
review. TrAC, Trends Anal. Chem..

[108] Norwitz N. G., Saif N., Ariza I. E., Isaacson R. S. (2021). Precision
nutrition for Alzheimer’s prevention in ApoE4 carriers. Nutrients.

[109] Lopes L. C., Santos A., Bueno P. R. (2022). An outlook on electrochemical
approaches for molecular diagnostics assays and discussions on the
limitations of miniaturized technologies for point-of-care devices. Sensor Actuator Rep..

